# Kiwifruit Genome Database (KGD): a comprehensive resource for kiwifruit genomics

**DOI:** 10.1038/s41438-020-0338-9

**Published:** 2020-08-01

**Authors:** Junyang Yue, Jiacheng Liu, Wei Tang, Ya Qing Wu, Xiaofeng Tang, Wei Li, Ying Yang, Lihuan Wang, Shengxiong Huang, Congbing Fang, Kun Zhao, Zhangjun Fei, Yongsheng Liu, Yi Zheng

**Affiliations:** 1 School of Horticulture, Anhui Agricultural University, Hefei, 230036 China; 2 School of Food and Biological Engineering, Hefei University of Technology, Hefei, 230009 China; 3 Boyce Thompson Institute, Cornell University, Ithaca, NY 14853 USA; 4 USDA-Agricultural Research Service, Robert W. Holley Center for Agriculture and Health, Ithaca, NY 14853 USA; 5 Ministry of Education Key Laboratory for Bio-resource and Eco-environment, College of Life Science, State Key Laboratory of Hydraulics and Mountain River Engineering, Sichuan University, Chengdu, 610064 China; 6 Beijing Advanced Innovation Center for Tree Breeding by Molecular Design, Beijing University of Agriculture, Beijing, 102206 China; 7 Plant Science and Technology College, Beijing University of Agriculture, Beijing, 102206 China

**Keywords:** Bioinformatics, Gene expression profiling, Plant breeding

## Abstract

Kiwifruit (*Actinidia* spp.) plants produce economically important fruits containing abundant, balanced phytonutrients with extraordinarily high vitamin C contents. Since the release of the first kiwifruit reference genome sequence in 2013, large volumes of genome and transcriptome data have been rapidly accumulated for a handful of kiwifruit species. To efficiently store, analyze, integrate, and disseminate these large-scale datasets to the research community, we constructed the Kiwifruit Genome Database (KGD; http://kiwifruitgenome.org/). The database currently contains all publicly available genome and gene sequences, gene annotations, biochemical pathways, transcriptome profiles derived from public RNA-Seq datasets, and comparative genomic analysis results such as syntenic blocks and homologous gene pairs between different kiwifruit genome assemblies. A set of user-friendly query interfaces, analysis tools and visualization modules have been implemented in KGD to facilitate translational and applied research in kiwifruit, which include JBrowse, a popular genome browser, and the NCBI BLAST sequence search tool. Other notable tools developed within KGD include a genome synteny viewer and tools for differential gene expression analysis as well as gene ontology (GO) term and pathway enrichment analysis.

## Introduction

Kiwifruit, belonging to the basal genus *Actinidia* within the family Actinidiaceae, consists of ~54 species and 75 taxa^1^. All species in this genus are perennial, deciduous and dioecious plants with a climbing or scrambling growth habit. They are native to southwestern China, although they are prevalent in New Zealand after being introduced in the early 20th century^2^. Despite the availability of diverse, rich germplasm resources for kiwifruit, only several economically important horticultural species have been successfully domesticated and widely cultivated, including *A. chinensis* Planchon, *A. deliciosa* (*A. chinensis* var. *deliciosa* A. Chevalier), *A. arguta* (Siebold and Zuccarini) Planchon ex Miquel and *A. eriantha* Bentham^3^.

Despite its relatively short history of domestication, kiwifruit has become an important fresh fruit worldwide. Owing to its remarkably high contents of vitamin C and minerals, kiwifruit is commonly celebrated as ‘the king of vitamin C’ and ‘the king of fruits’. In addition to being primarily consumed as fresh fruits, kiwifruit can be used for medicinal purposes (as observed for the fruits and roots) and for its ornamental value (as observed for the flowers)^4^. Furthermore, kiwifruit provides a distinct model system for studies of several fundamental biological processes, such as ascorbic acid biosynthesis^5^ and sex determination^6,7^.

To facilitate genetic research and molecular breeding in kiwifruit, in 2013, the International Kiwifruit Genome Consortium assembled and published the first reference kiwifruit genome for the Chinese cultivar *A. chinensis* ‘Hongyang’ (2*n* = 2× = 58)^8^. This genome sequence, along with its structural and functional annotations, was subsequently released online at the Kiwifruit Information Resource (KIR; http://kir.atcgn.com/)^9^. KIR has played a vital role by providing the scientific community with access to the ‘Hongyang’ genome sequence and associated annotation data. However, KIR also meets new requirements for broader utility as large volumes of genome and transcriptome data have been generated at an increasingly rate during the past few years, especially in the form of two recently published kiwifruit genome assemblies from *A. chinensis* ‘Red5’^10^ and *A. eriantha* ‘White’^11^ and an improved assembly of ‘Hongyang’^12^. Moreover, a number of transcriptome studies have been recently reported in various kiwifruit species, including *A. arguta, A. chinensis, A. deliciosa*, and *A. eriantha*^13–15^. Therefore, there is an urgent need for a central and integrated database to store, analyze, mine, manage and disseminate these large-scale datasets for the kiwifruit research and breeding community.

For this purpose, we rebuilt and describe herein an updated kiwifruit genome database (KGD; http://kiwifruitgenome.org/), which currently integrates rich genome and transcriptome resources of kiwifruit, including assembled genomes and predicted gene models. At the same time, we performed comprehensive functional annotations for these gene models, identified conserved syntenic genome blocks between different kiwifruit species, and incorporated gene expression profiles based on publicly available RNA-Seq datasets. KGD was constructed using the Tripal system^16^, a specific toolkit for the construction of online community genomic databases, by integrating the GMOD Chado database schema^17^ and the Drupal open source platform (https://www.drupal.org/). Furthermore, a set of modules and user-friendly interfaces have been implemented in KGD to analyze and visualize comparative genomic and transcriptome profiling datasets for different kiwifruit species.

## Database content

### Genome sequences

High-quality genome sequences of three kiwifruit cultivars have been assembled to date, including two from *A. chinensis* (‘Hongyang’ and ‘Red5’)^8,10,12^ and one from *A. eriantha* (‘White’)^11^. For the ‘Hongyang’ cultivar, there are two versions (2.0 and 3.0) of the genome assembly, among which version 3.0 was assembled using PacBio long reads^12^ and thus shows much higher genome contiguity and sequence quality than version 2.0, which was assembled purely from Illumina short reads^8^. The genome, transcript and protein sequences of the predicted protein-coding genes and the gene annotation files in GFF3 format were downloaded from the Kiwifruit Information Resource^9^ (ftp://www.atcgn.com/kiwifruit/) for ‘Hongyang’ version 2.0 and ‘White’, from 10.6084/m9.figshare.10046558 for ‘Hongyang’ version 3.0, and from the Ensemble database (https://plants.ensembl.org) for ‘Red5’.

### Functional annotation of protein-coding genes

A total of 156,257 protein-coding genes were predicted from these four genome assemblies, including 39,761 from ‘Hongyang’ version 2.0, 40,464 from ‘Hongyang’ version 3.0, 33,044 from ‘Red5’, and 42,988 from ‘White’. A standard, unified procedure was used to comprehensively annotate the predicted protein-coding genes. First, the protein sequences of the predicted genes were aligned against the NCBI non-redundant (nr), UniProt (Swiss-Prot and TrEMBL), and Arabidopsis protein (TAIR) databases using the BLASTP command of DIAMOND^18^ with an E-value cutoff of 1e-5. All of these protein sequences were further compared against the InterPro database using InterProScan^19^ to identify functional domains. The BLASTP results derived from the nr database and the identified InterPro domains were fed into the Blast2GO pipeline^20^ to assign gene ontology (GO) terms to each protein-coding gene. The BLASTP results against the UniProt and TAIR databases were fed to the AHRD program (https://github.com/groupschoof/AHRD) to obtain concise, precise and informative gene function descriptions. We also used the PathwayTools program^21^ to predict biochemical pathways encoded by each of the kiwifruit genomes. For each genome, the gene function descriptions derived from the AHRD analysis, the GO terms assigned by the Blast2GO tool, and the enzyme commission (EC) numbers collected from the UniProt database were integrated into a single file in PathoLogic format, which was directly used by PathwayTools for pathway prediction. In total, 342 to 405 predicted biochemical pathways were obtained from each of these four kiwifruit genomes.

### Comparative genomic analysis

We identified syntenic blocks and homologous gene pairs within syntenic blocks in the four kiwifruit genome sequences, including comparisons both within each genome and between any two genomes. The protein sequences were first aligned against themselves (within each genome) as well as between each other (pairwise comparisons) using BLASTP^22^ with an E-value cutoff of 1e-5 and a maximum of five alignments. Based on the BLASTP results and gene positions, syntenic blocks were determined using MCScanX^23^ with default parameters. A total of 14,125 syntenic blocks and 335,140 homologous gene pairs were identified, among which approximately 800–1100 syntenic blocks and 15,000–20,000 homologous gene pairs were identified within each genome, and 1500–2700 and 48,000–55,000 were identified between any two of the four genomes.

### Gene expression profiles

We collected all publicly available kiwifruit RNA-Seq datasets from the NCBI Sequence Read Archive (SRA) database, including data from nine projects and 80 samples. Most of these samples were derived from fruits (35 samples), dormant buds (17 samples), and phloem tissues (10 samples), and others were derived from leaves, seedlings, roots and stems. A unified pipeline was applied to process and analyze these RNA-Seq datasets. Briefly, raw RNA-Seq reads were processed to remove adaptor and low-quality sequences using Trimmomatic^24^. Trimmed reads shorter than 80% of their original length were discarded. The remaining cleaned reads were then aligned against the SILVA rRNA database^25^ using the Bowtie program (version 1.1.2)^26^ allowing up to three mismatches, and the mapped reads were removed. The resulting high-quality reads were aligned to the kiwifruit genomes using the STAR program^27^ with a maximum of two mismatches. Finally, based on the alignments, the read counts of each gene were calculated and normalized to FPKM (fragments per kilobase of transcripts per million mapped fragments) values. The mean and standard error of the FPKM values of the biological replicates were then derived.

### Transcription factors and transcriptional regulators

We used the iTAK program^28^ to identify transcription factors (TFs) and transcriptional regulators (TRs) from the four kiwifruit genomes and classified them into different families. The protein sequences of the predicted protein-coding genes were fed into iTAK for TF and TR identification and classification with the default parameters. A total of 9906 TFs (2323–2718 from each genome) belonging to 54 different families and 2211 TRs (533–563 from each genome) belonging to 25 different families were identified.

## Database implementation

The Tripal system^16^ was employed to facilitate the construction of KGD. Tripal provides dozens of extension models for building online genomic databases. The genome sequences, predicted gene models, mRNA and protein sequences were loaded into the database using the ‘Data Loaders’ function of Tripal. For gene functional annotations, the top BLASTP hits as well as the GO terms and InterPro domains assigned to each gene were imported into KGD through Tripal Analysis Extension Modules. The functional descriptions generated by the AHRD program were loaded into KGD using an in-house Perl script. Additionally, TFs and TRs were imported into KGD using the gene family extension module that we developed previously.

**Fig. 1:**
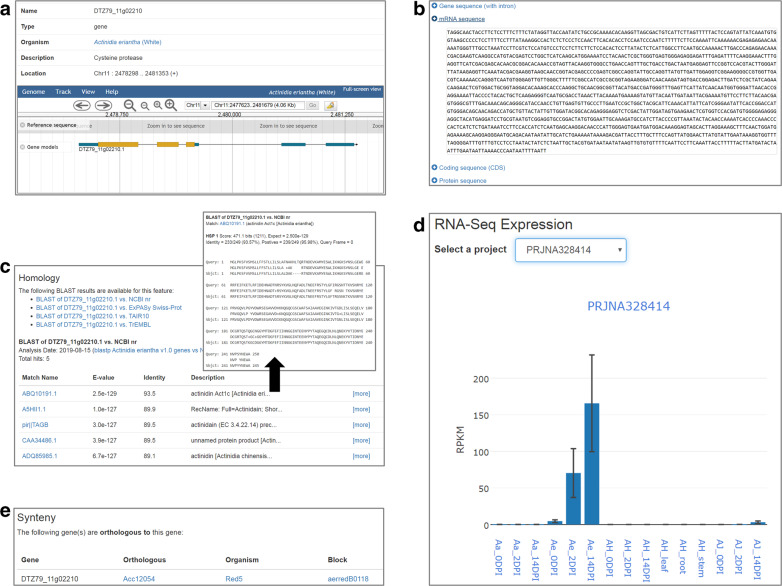
Gene feature page in KGD. The page contains different sections with different content types, including (**a**) overview of information for the gene (gene position, structure, and functional annotation), (**b**) gene/mRNA/protein sequences, (**c**) homologous genes and sequence alignments generated by BLAST, (**d**) RNA-Seq expression profiles, and (**e**) synteny blocks related to the gene

KGD provides a page for each kiwifruit genome assembly, typically comprising multiple categories of biological information, and submenus to access data analysis tools including tools for performing queries, BLAST searches, genome browsing, pathway analysis, and downloads of the genome resources. KGD also generates a page for each queried gene (gene feature page) that includes categories of basic information and the gene structure displayed in a genome browser (Fig. 1a), genome/mRNA/protein sequences (Fig. 1b), functional annotations and homologous genes (Fig. 1c), expression profiles (Fig. 1d), and syntenic blocks (Fig. 1e).

To import the expression information (read counts and FPKM values) as well as the corresponding experimental metadata into KGD, we used two Tripal extension modules: ‘SRA’ and ‘RNA-Seq’, which we previously developed^29^. The ‘SRA’ extension module is a mimic of the NCBI SRA database for the purpose of managing the meta-information of projects, samples, and experiments but does not require the storage of raw reads. The ‘RNA-Seq’ module is designed to manage and display gene expression profiles. In KGD, the ‘RNA-Seq’ home page lists all collected projects and provides mouse-over descriptions in which after an RNA-Seq project is selected, the meta-information of the project is displayed. Furthermore, a submenu including the ‘Heatmap’, ‘DEGs’ and ‘Expression Viewer’ is provided to guide users to explore and analyze the expression datasets. Additionally, gene expression profiles can be accessed under the ‘RNA-Seq Expression’ section within the gene feature page (Fig. 1d).

The identified syntenic blocks and homologous gene pairs were loaded into KGD using the ‘SyntenyViewer’ module. The ‘Synteny’ section on the gene feature page has been designed to display all available syntenic blocks and homologous gene pairs associated with a specific gene (Fig. 1e). Furthermore, each syntenic block can be linked to a new page that lists all genes located in the syntenic region.

A biochemical pathway database for different *Actinidia* species, ActCyc, was implemented within KGD using the PathwayTools web server^21^. Through ActCyc (http://kiwifruitgenome.org/pathway), users can easily search biochemical pathways and perform comparative analyses.

## Utility and discussion

### Query option

In summary, two search categories are provided in KGD: gene search and batch query. The gene search option provides an interface for querying KGD with a gene ID or keyword associated with gene annotations. To facilitate the queries of genes and functional annotation data stored in KGD, we employed the Apache Solr search engine (http://lucene.apache.org/solr/) to build indexes for different sources of annotation information, including gene functions, GO terms, InterPro domains and homologs.

**Fig. 2:**
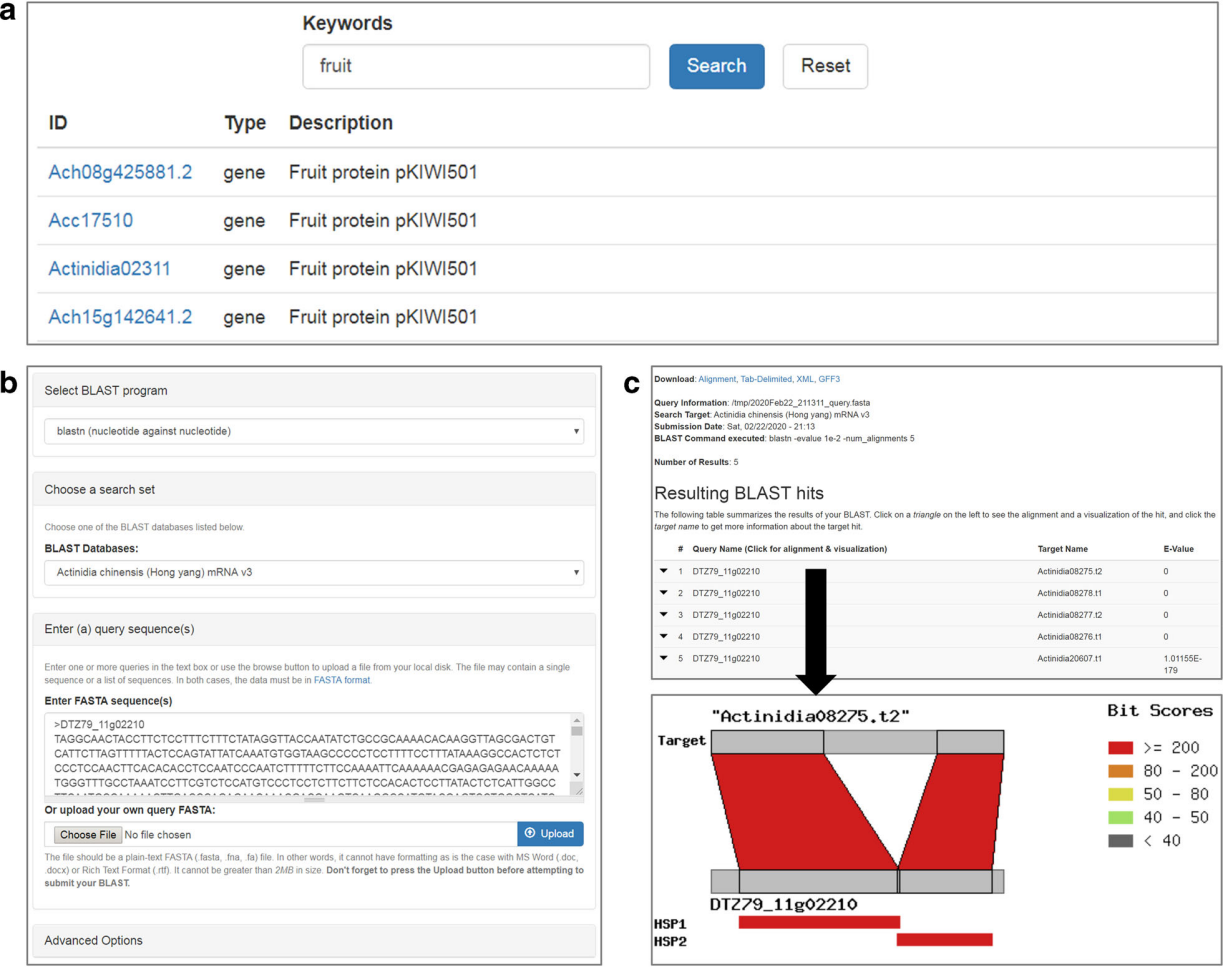
Search functions in KGD. **a** List of genes returned from a global search using a keyword. **b** Interface of the homology search (BLAST) implemented in KGD. **c** Result page of the homology search. The bottom image illustrates the alignment of query and subject sequences

In addition to the gene search option under each genome page, a global search function is provided under the main menu of KGD. This function provides a quick query against all the records stored in the database and returns results in a tabular format including the gene ID, gene type, and gene description (Fig. 2a). From this table, users can browse the detailed feature page for each gene by clicking the corresponding gene link.

The batch query option allows users to retrieve sequences, annotations and other types of information (e.g., TFs and TRs) for a given list of genes. The batch query function in KGD was modified from the ‘Sequence Retrieval’ page of Tripal^16^.

### Homology search

To provide a homology search function, we implemented the Tripal BLAST UI extension module in KGD. All genome, mRNA, CDS and protein sequences of kiwifruit species stored in KGD are available for comparison through the BLAST program. To prevent users from selecting inompatible BLAST programs (BLASTN, BLASTP, BLASTX, tBLASTN and tBLASTX) for the corresponding databases, the list of BLAST programs is automatically set up according to the selected reference database (Fig. 2b). Options for filtering low-complexity sequences and selecting the maximum number of returned BLAST hits are provided. The BLAST function provides downloadable output files ordered by the expected values in three different formats, HTML, TSV and XML, and the results page lists all the hits, with each hit linked to a graphic output that shows the alignment coordinates between the query and the hit and a color-ranked bit score for the alignment (Fig. 2c).

### Genome browser

In KGD, we implemented JBrowse^30^, a widely used genome browser, to display genome sequences, gene models, and expression profiles. Currently, all publicly available kiwifruit genomes, predicted gene models, and gene expression profiles derived from RNA-Seq data have been imported into JBrowse. The tracks of a given gene in a reference genome are also embedded in the gene features page to provide a graphical and informative view of its sequence and structure (Fig. 1a). Additionally, the genome browser can support other types of interesting data, such as single-base resolution genome variants, when they become available in the near future.

### Synteny viewer

**Fig. 3:**
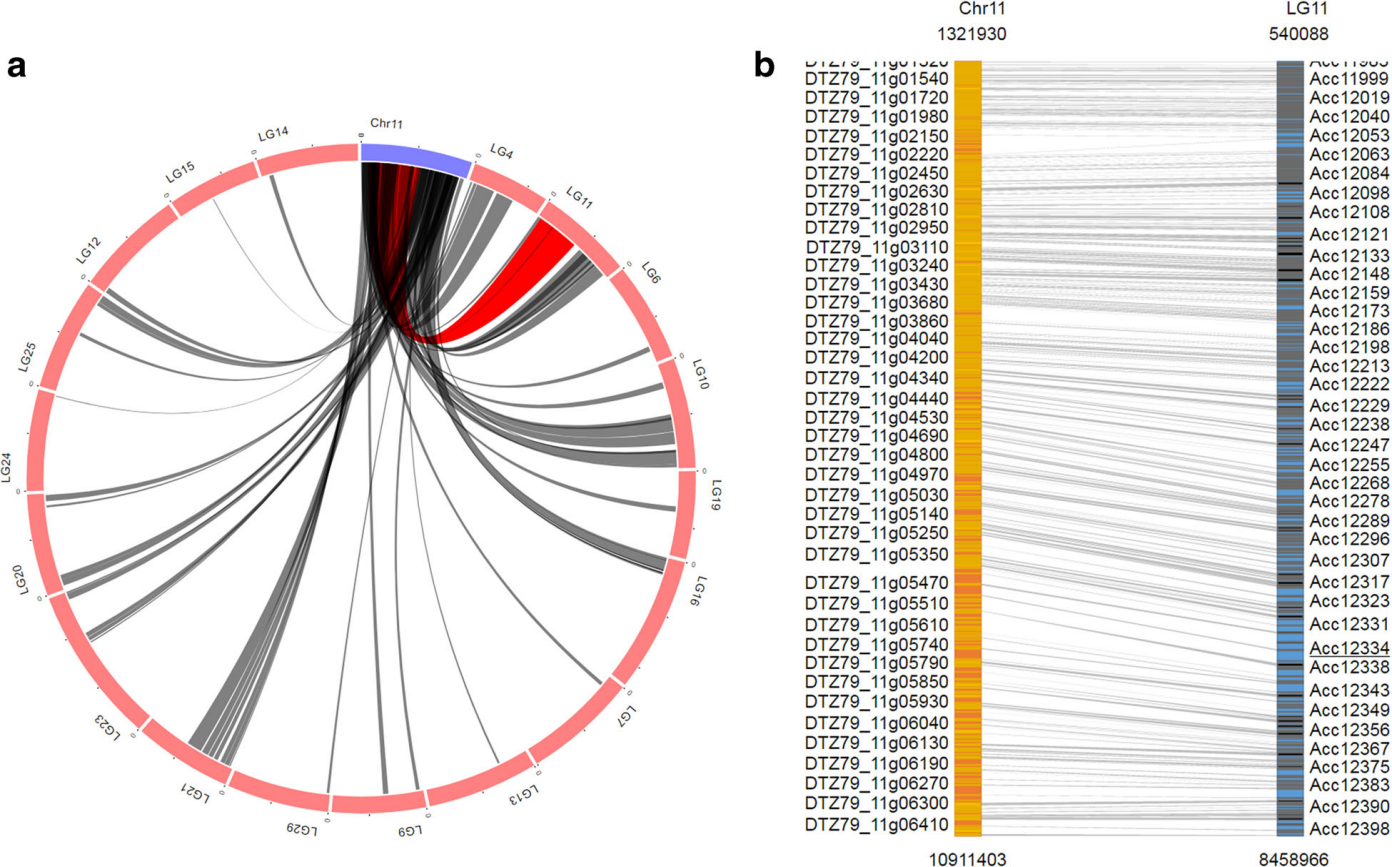
Genome synteny viewer in KGD. **a** Syntenic blocks displayed in a Circos plot. The blue arc indicates the query chromosome, and the red arcs indicate the chromosomes of the compared genome. Gray lines between blue and red arcs indicate syntenic blocks identified between the two genomes. The lines of a syntenic block will become red when the user mouses over it. **b** Detailed view of a specific synteny block. The query and compared chromosomes of a specific synteny block are shown in orange and blue, respectively. The yellow and black lines within each chromosome indicate homologous gene pairs, which are connected by gray lines

To view syntenic blocks and homologous gene pairs between different kiwifruit genome assemblies, we developed ‘SyntenyViewer’, an extension module of Tripal, in KGD. Syntenic blocks can be retrieved by selecting a query genome together with one or more subject genomes. ‘SyntenyViewer’ will draw circus plots to display syntenic blocks for every pair of query and subject genomes (Fig. 3a) and simultaneously generate a full list of the syntenic blocks. For a specific syntenic block, ‘SyntenyViewer’ creates an image to display the homologous gene pairs, and the view can be zoomed in or out as desired (Fig. 3b). The full list of genes included in the homologous gene pairs is provided with links to the detailed feature page of each gene (Fig. 1). In brief, the ‘SyntenyViewer’ module can not only reveal syntenic blocks between any two genome sequences but also connect homologous gene pairs in syntenic blocks. With this module, homologous members of interesting genes that are located in a specific region of one kiwifruit genome can be easily identified and intuitively viewed for the other kiwifruit genome.

### Enrichment analysis

Large-scale genomic studies typically result in large lists of interesting genes. Interpreting such gene lists to obtain biologically meaningful information is the basic premise for understanding the underlying regulatory mechanisms of important biological processes and biochemical pathways. Enrichment analysis is a powerful and frequently used method for identifying specific families or groups of genes that are overrepresented in a list of biological entries (e.g., GO terms and biochemical pathways). We previously developed two custom-built extension modules of Tripal, ‘GO tool’ and ‘Pathway tool’, based on the hypergeometric test^29^. These two modules were also implemented in KGD to identify significantly enriched GO terms and biochemical pathways from a list of user-provided genes.

### RNA-Seq expression analysis

**Fig. 4:**
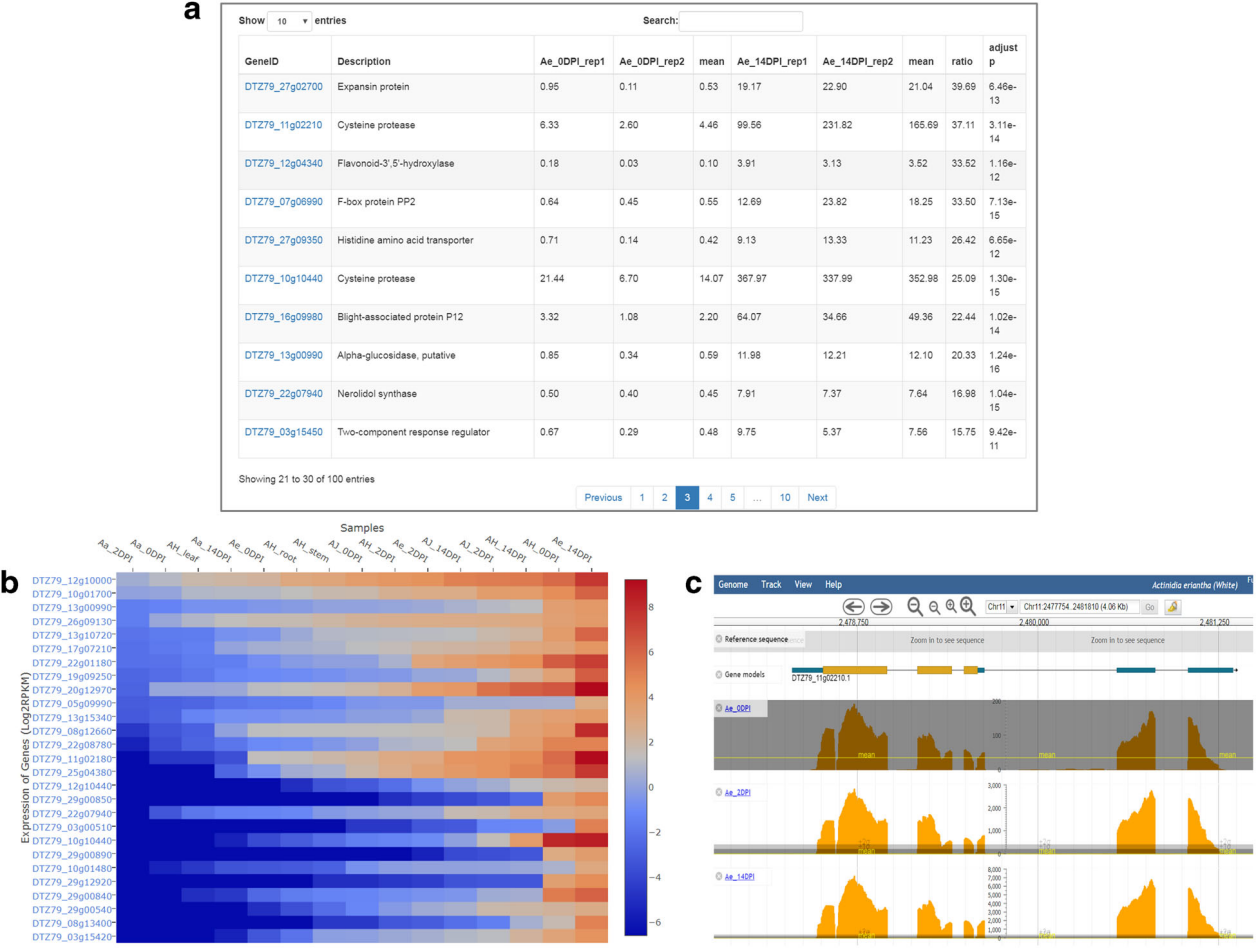
Gene expression analysis with the ‘RNA-Seq’ module in KGD. **a** Statistical analysis results listing the top 100 DEGs ordered by adjusted *p*-values. **b** Heatmap showing the expression profiles of a list of user-defined genes. **c** Single-base resolution expression profile view in JBrowse

KGD not only stores gene expression profiles derived from RNA-Seq datasets but also provides an ‘RNA-Seq’ module to allow users to perform RNA-Seq data analyses, including the identification of differentially expressed genes (DEGs) and the visualization of gene expression profiles. The two most popular DEG identification tools, edgeR^31^ and DESeq^32^, were integrated into the ‘RNA-Seq’ module in KGD. The tools provide users the option of selecting their desired cutoff values for the gene expression fold change and adjusted *P*-value to determine the final DEGs. The result page for the DEG analysis includes the project description, parameter settings, top 100 DEGs ordered by adjusted *P*-values, and a download link to a file with all identified DEGs together with their relevant information (Fig. 4a). Furthermore, the result page provides links to other modules for many downstream analyses of the identified DEGs, such as BLAST, batch query, GO term and pathway enrichment analyses, and gene functional classification.

In addition to viewing the expression profiles of individual genes on the gene feature page (Fig. 1d), the ‘RNA-Seq’ module provides two interactive visualization tools: a heatmap tool developed using Plotly’s JavaScript library (http://plot.ly) for displaying the expression profiles of a set of genes (Fig. 4b) and an expression viewer embedded in JBrowse for displaying single-base resolution expression profiles under certain conditions (Fig. 4c).

## Conclusion and future directions

We have constructed the KGD, which serves as a central portal for kiwifruit genomics and provides comprehensive genome and transcriptome resources for kiwifruits. KGD stores the sequences of various kiwifruit genome assemblies, predicted mRNAs and proteins as well as comprehensive functional annotations, genome synteny blocks, homologous gene pairs, gene expression profiles, and biochemical pathways. The database also offers various query, analysis and visualization tools, including tools for basic and batch queries, BLAST, a genome browser, a biochemical pathway database (ActCyc), tools for GO term and pathway enrichment analysis, a genome synteny viewer and a DEG analysis tool. An important feature of KGD is that four modules recently developed by our groups, a ‘GO tool’, ‘Pathway tool’, ‘SyntenyViewer’ and ‘RNA-Seq’, have been implemented to extend the capabilities of the database.

KGD will be continuously updated when new genome, transcriptome and other types of genetic datasets of kiwifruit species become publicly available. Additionally, we will continue to develop novel extension modules that can be adopted by the Tripal community. We believe that KGD will be a global, active platform for researchers and breeders working with kiwifruit as well as other plant species.
